# Modulation of fibronectin and laminin expression by Rhodium (II) citrate-coated maghemite nanoparticles in mice bearing breast tumor

**DOI:** 10.1038/s41598-017-18204-1

**Published:** 2017-12-20

**Authors:** Márcia Rocha, Rachel Arcanjo, Cláudio Lopes, Marcella Carneiro, Aparecido Souza, Sônia Báo

**Affiliations:** 1 Nanoscience and Nanotechnology Postgraduate Program, University of Brasília, 70.910-900 Brasília, DF Brazil; 2 Institute of Biological Sciences, University of Brasília, 70.910-900 Brasília, DF Brazil; 3 Institute of Chemistry, Federal University of Goiás, 74.690-900 Goiás, GO Brazil

## Abstract

Degradation of cellular matrix is one of the important processes related to the progression of breast cancer. Tumor cells have the ability to exhibit necessary conditions for growth and survival, promoting degradation processes of extracellular matrix proteins, such as laminin (LN) and fibronectin (FN). In this study, we evaluated whether treatments, based on free rhodium (II) citrate (Rh_2_(H_2_cit)_4_), maghemite nanoparticles coated with citrate (Magh-cit) and maghemite nanoparticles coated with rhodium (II) citrate (Magh-Rh_2_(H_2_cit)_4_), in murine metastatic breast carcinoma models can modulate the expression of laminin and fibronectin proteins. Synthesized nanoparticles were characterized using X-ray diffraction, transmission electron microscopy, energy dispersive spectroscopy and dynamic light scattering. The expression of FN and LN was assessed using immunohistochemistry and western blotting. The gene expression of FN1 and LAMA1 were evaluated using real-time PCR. The FN1 and LAMA1 transcripts from the Magh-Rh_2_(H_2_cit)_4_ treated group were 95% and 94%, respectively, lower than the control group. Significant reduction in tumor volume for animals treated with Magh-Rh_2_(H_2_cit)_4_ was observed, of about 83%. We witnessed statistically significant reductions of FN and LN expression following treatment with Magh-Rh_2_(H_2_cit)_4_. We have demonstrated that the antitumor effects of Magh-Rh_2_(H_2_cit)_4_ and Rh_2_(H_2_cit)_4_ regulate the expression of FN and LN in metastatic breast tumors.

## Introduction

The extracellular matrix (ECM) is a structure that influences and regulates some primordial aspects of cell biology such as differentiation, proliferation, migration, and modulates cell adhesion^1^. Some components of ECM are insoluble proteins (i.e. fibronectins, laminins, collagens and elastin), proteoglycans, growth factors, small matricellular proteins and small integrin-binding glycoproteins^2^. In cancer, the ECM plays a central role in the progression of the disease. Cells such as fibroblasts contribute to tumor growth and survival. During disease progression, some properties of ECM are altered including deposition of proteins, reorganization, composition, structure and rigidity. The malignancy of a tumor can be related to alterations both in ECM and tumor cells, or to degradation and synthesis of ECM components^3^. In this context, laminin and fibronectin have been shown to play an important role in tumor invasion. Studies suggest a correlation between laminin and fibronectin receptor expression in tumor cells and tumor progression^4,5^.

Fibronectin (FN) is a heterodimeric glycoprotein that can be found in the ECM. This protein can be synthesized as a dimer with two subunits (~250 kDa), and each monomer has three types of domains (FNI, FNII and FNIII), with affinity for many ECM proteins, cell surface integrin receptors, heparin and sulfate moieties^6^. FN can be found in two forms: plasmatic (soluble) and cellular (insoluble). The plasmatic form is synthesized principally by hepatocytes which circulate in the bloodstream, while the cellular FN is produced by mesenchymal and epithelial cells that deposit insoluble fibers in the ECM of connective tissues^7^. FN plays a role in adhesion (cell-cell and cell-matrix), differentiation, migration, oncogene transformation, growth and proliferation^8^. Studies showed that FN can have a modulating effect in tumors showing different expression and deposition levels as compared with normal tissue. This is important because tumor progression is mediated by altered ECM^9^. Thus, understanding the dynamics of FN in tumorigenesis is essential to elucidate the mechanisms of cancer progression.

Laminin (LN) is a large heterotrimeric and non-collagenous glycoprotein of basement membrane^10^. LN have three subunits (β, α and γ), and their combinations assemble 14 laminin isoforms that have several functions and different tissue distributions^11^. Important biological functions of LN isoforms have been described, as maintenance and survival^10,11^; adhesion^12^; differentiation^13^; migration^14^; cell proliferation^12,14^; control of gene expression^15^; angiogenesis and metastasis^11,15^. The interaction of laminin with tumor cells increases their metastatic potential. Some of the mechanisms that laminin uses to promote tumor dissemination are the induction of proteases that degrade components of ECM and tumor cell proliferation^12,14^.

Drug Delivery Systems, on a nanometer scale, can improve the effectiveness of cancer treatments. These systems have advantages when compared to conventional therapies such as, increased efficacy, progressive and controlled drug release, reduction of treatment toxicity, prolonged time in blood circulation, and reduced number of doses and targeting^16^. Nanoparticles that are used for biological applications require surface modifications to make them biocompatible, non-aggregable, non-toxic and stable^17^. Iron oxide nanoparticles, such as maghemite (γ-Fe_2_O_3_), are one the most widely used in biological applications^18^. A compound that has being used for surface modification of nanoparticles is the rhodium (II) citrate (Rh_2_(H_2_cit)_4_), an analogue of cisplatin, which displays cytotoxic, cytostatic and antitumor activity in mammary carcinoma cells. Therefore, the association of rhodium (II) citrate with maghemite nanoparticles (Magh-Rh_2_(H_2_cit)_4_) and maghemite nanoparticles coated with citrate (Magh-cit) is a strategy employed in an attempt to reduce toxicity in the organism and increase specificity in the target tissue during cancer treatment^19^.

This study aims to elucidate the profile of FN and LN expression in invasive breast tumors (4T1) and their role in tumor progression. The FN and LN expression was correlated with the efficacy of treatment (tumor volume) after Intratumoral administration of Magh-Rh_2_(H_2_cit)_4_ or Magh-cit or Rh_2_(H_2_cit)_4_. We also evaluated the antitumor activity induced by these treatments in Balb/c mice bearing 4T1 breast tumors.

## Results and Discussion

### Nanoparticles characterization

**Figure 1:**
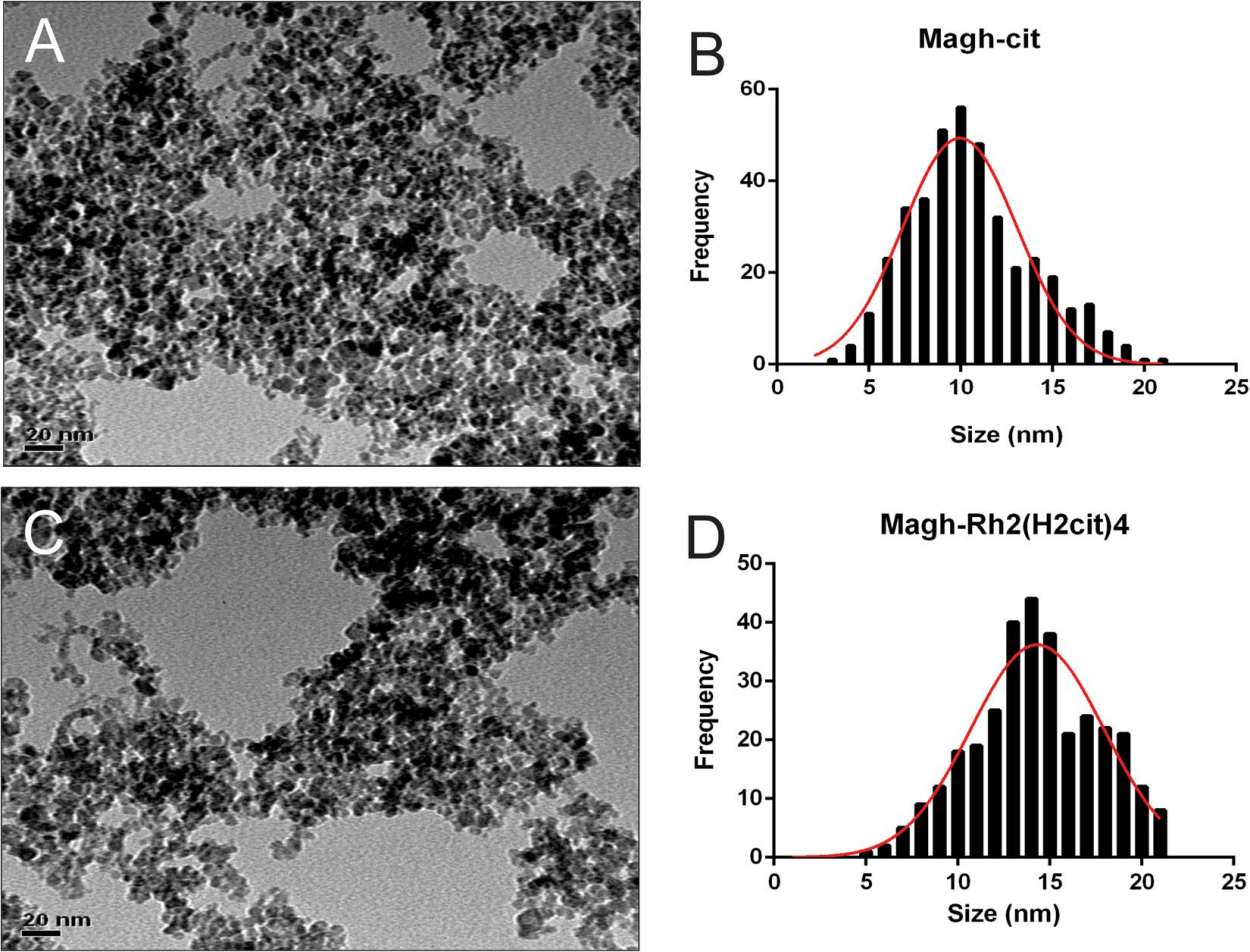
Transmission electron microscopy (TEM) micrographs of Magh-cit (**A**) and Magh-Rh_2_(H_2_cit)_4_ (**C**) nanoparticles at a magnification of 400 K. Particle size distribution for Magh-cit (**B**) and Magh-Rh_2_(H_2_cit)_4_ (**D**).

The particle size and morphology of Magh-cit and Magh-Rh_2_(H_2_cit)_4_ nanoparticles were evaluated using transmission electron microscopy (TEM). Figure 1A shows that Magh-cit nanoparticles are spherical with a mean diameter of 10 nm (Fig. 1B). After coating with rhodium citrate, the mean diameter increased to about 14 nm (Fig. 1B–D). These results demonstrate that the coating process is not significant to agglomerate and to change the size of the particles.

In X-ray diffractometry (XRD) analysis, it was shown same peaks characteristic with standard γFe_2_O_3_ (JCPDS card No. 39-1346) and without other crystalline phases detected in Magh-cit and Magh-Rh_2_(H_2_cit)_4_ samples (see Fig. S1 in the Supplementary information). The crystallite size of Magh-cit and Magh-Rh_2_(H_2_cit)_4_ was about 6.8 nm and 14 nm, respectively. It was measured from the XRD pattern according to Scherrer formula at the 311 peak.

As shown in Figures S2 (see in the Supplementary information), energy dispersive spectroscopy (EDS) as a function of element and probe position for the line scan reveals in the marked area that only iron (Fe,86.14%) and Oxygen (O, 13.86%) elements are detected in the Magh-cit sample (Fig. S2-A), while in the Magh-Rh_2_(H_2_cit)_4_ sample was shown iron (Fe, 55.15%) rhodium (Rh, 6.78%) Oxygen (O, 36.62%) and Carbon (C, 1.46%) could be identified (Fig. S2-B). These results confirm that there is no contamination in the synthesis of the samples and corroborates with the data found in the XRD.

The samples Magh-cit and Magh-Rh_2_(H_2_cit)_4_ presented a negative zeta potential at room temperature of about −30 mV and −38 mV, respectively. This negative charge indicates an electrostatic stabilization of the particles. The mean hydrodynamic particle size was about 79 nm for Magh-cit and 137 nm for Magh-Rh_2_(H_2_cit)_4_. The size distribution was polymodal and the polydispersion index (PDI) was between 0.1 and 0.2.

In order to assess the effect of variations in temperature on the stability of samples, was did heating-cooling cycles were carried out followed by hydrodynamic particle size measurements. Six cycles of 4 C° and 37 °C with storage at each temperature for 24 hours were performed. After subject to this stress test, samples remained stable in all temperatures observed. There were no statistically significant differences in these results (Tables 1 and 2).

**Table 1:** Data of the Hydrodynamic diameter of the Magh-cit subjected to the heating–cooling cycles.

37 °C				4 °C			
**Cycle**	**Size (nm)**	**Zeta (mV)**	**PDI**	**Cycle**	**Size (nm)**	**Zeta (mV)**	**PDI**
1	67.1 ± 0.1	−32.8 ± 0.01	0.2 ± 1.68	2	79.3 ± 0.6	−32.8 ± 2.7	0.2 ± 0.01
3	61.1 ± 0.2	−31.5 ± 0.01	0.2 ± 0.01	4	73.3 ± 0.7	−33.8 ± 1.3	0.2 ± 0.06
5	63.6 ± 0.2	−32.2 ± 0.01	0.2 ± 1.45	6	72.8 ± 0.3	−33.5 ± 1.1	0.2 ± 0.01
**Mean**	63.9 ± 0.1	−32.1 ± 0.01	0.2 ± 1.04	**Mean**	75.1 ± 0.7	−33.3 ± 1.7	0.2 ± 0.02

**Table 2:** Data of the Hydrodynamic diameter of the Magh-Rh_2_(H_2_cit)_4_ nanoparticles subjected to the heating–cooling cycles.

37 °C				4 °C			
**Cycle**	**Size (nm)**	**Zeta (mV)**	**PDI**	**Cycle**	**Size (nm)**	**Zeta (mV)**	**PDI**
1	185.2 ± 1.8	−37.3 ± 0.2	0.2 ± 0.01	2	136.3 ± 1.6	−38.5 ± 0.3	0.1 ± 0.01
3	189.6 ± 0.6	−33.4 ± 0.2	0.2 ± 0.02	4	146.4 ± 1.2	−37.8 ± 0.2	0.1 ± 0.06
5	177.2 ± 1.6	−37.5 ± 0.2	0.2 ± 0.01	6	144.4 ± 0.2	−37.4 ± 0.1	0.1 ± 0.02
**Mean**	184.0 ± 1.3	−36.0 ± 0.2	0.2 ± 0.01	**Mean**	142.3 ± 0.6	−37.9 ± 0.2	0.1 ± 0.03

### Clinical aspects

**Figure 2:**
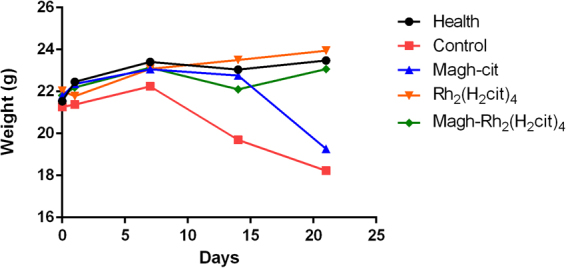
Mean weight of Balb/c mice bearing 4T1 breast tumor, control and health at the beginning and during all of treatment. The animals were treated with Rh_2_(H_2_cit)_4_, Magh-cit or Magh-Rh_2_(H_2_cit)_4_. No statistical significant changes were observed. ANOVA p > 0.05.

No behavioral or clinical changes were observed such as diarrhea, hair loss or reduced motor activity throughout the experiment for all studied groups, indicating a good level of tolerance by mice to the treatments. These results were also observed in previous studies done by our group^20^. The animals body weight was recorded prior to the beginning of treatment and, additionally, every week throughout its duration, as it is an important indicator of toxicity^21^ (Fig. 2).

Mean body weight of animals in the control group showed a reduction after 14 days of treatment, and animals of the group treated with Magh-cit displayed a reduction after the last day of treatment (21) but no statistical significant differences were observed. The administered doses did not seem to present any toxicity in the treated animals, as compared to the healthy group.

The hematological and biochemical parameters were also analyzed. The toxicity associated with cancer therapies often results in the emergence of some clinical signs such as anemia, infections, neutropenia, among others. Because of this, the evaluation of blood cells is done routinely in animal research. No significant differences were observed in the analyzed hematological parameters when compared with the control group (see Table S1 in the Supplementary information).

High plasma levels of creatinine and glutamic pyruvic transaminase (GPT) indicate renal overload and hepatic injury caused by the use of toxic drugs or infections, respectively^22^. In our study, no significant differences were observed in these biochemical parameters, suggesting that the treatments used may not cause renal overload, renal dysfunction and hepatic injury (see Table S1 in the Supplementary information).

### Antitumor effect in tumor-bearing mice and survival indices

**Figure 3:**
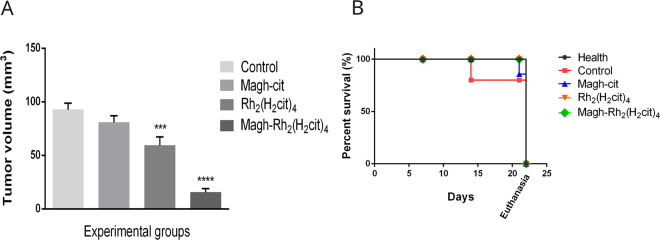
Antitumor effect in tumor bearing mice and survival indices. The mice were treated with five doses of Rh_2_(H_2_cit)_4_ (8 mg/kg) or Magh-cit (216 mg/kg iron) or Magh-Rh_2_(H2cit)_4_ (8 mg/kg of rhodium citrate and 216 mg/kg iron) until 21st day after tumor inoculation. (**A**) Regression of tumor volume of experimental groups mice. (**B**) Survival curve for tumor-bearing mice. The days are referenced from the beginning of the experiment to euthanasia. The values shown are the mean ± SEM ***p < 0.001, ****p < 0.0001.

To demonstrate the antitumor efficacy of Rh_2_(H_2_cit)_4_ (8 mg/kg), Magh-Rh_2_(H_2_cit)_4_ (8 mg/kg of rhodium (II) citrate and 216 mg/kg of iron) and Magh-cit (216 mg/kg of iron), these compounds were injected intratumorally every two days for Balb/c female mice with breast cancer. An accentuated decrease in tumor volume was observed in the treated groups. As shown in Fig. 3A, the Magh-Rh_2_(H_2_cit)_4_-treated group had a statistically significant reduction in tumor volume, of about 83%, and the group treated with Rh_2_(H_2_cit)_4_ had a statistically significant reduction of about 36%, when compared with the control group. These results demonstrate the antitumor efficacy of the compounds used in metastatic breast tumors. This higher antitumor effect of Magh-Rh_2_(H_2_cit)_4_ treatment, using nanoparticles, is most likely due to their weak lymphatic drainage in the tumor region and heterogeneous vascularization with fenestrated vessels that allow for the easy passage of nanoparticles and their retention in the tumor. Other characteristics such as their association with rhodium complex, their size and the intratumor administration of treatment can also be associated with this result. Carneiro *et al*.^20^ investigating the antitumor effect of Magh-Rh_2_(H_2_cit)_4_ under the same conditions of our study, found a significant reduction of tumor volume in Balb/c mice.

Additionally, we observed the survival curve of animals during treatments (Fig. 3B). All mice treated with Rh_2_(H_2_cit)_4_ and Magh-Rh_2_(H_2_cit)_4_ nanoparticles survived until the end of the experiment. In the group treated with Magh-cit nanoparticles, one mouse died after 21 days and one mice from the control group that did not receive treatment died after 14 days of experimentation (blue and red lines, respectively). This data suggests that rhodium in its free form, or associated with nanoparticles has the ability to enhance survival rates, drug targeting and toxicity reduction in normal cells. In this way, we show that nanocarriers can improve antitumor effects and reduce the toxicity of anticancer drugs.

### Immunohistochemistry analysis

The ECM exert effects on cell behavior and may facilitate tumor progression. In addition, changes in the expression of specific components of ECM such as fibronectin and laminin have been associated with a worse prognosis for patients with breast cancer. It is important, therefore, to identify markers that can elucidate the behavior of the tumor, especially in breast cancer due to the variability of clinical progression of the disease. In breast cancer, the expression and distribution of FN are altered when compared with normal breasts^6,23,24^.

**Figure 4:**
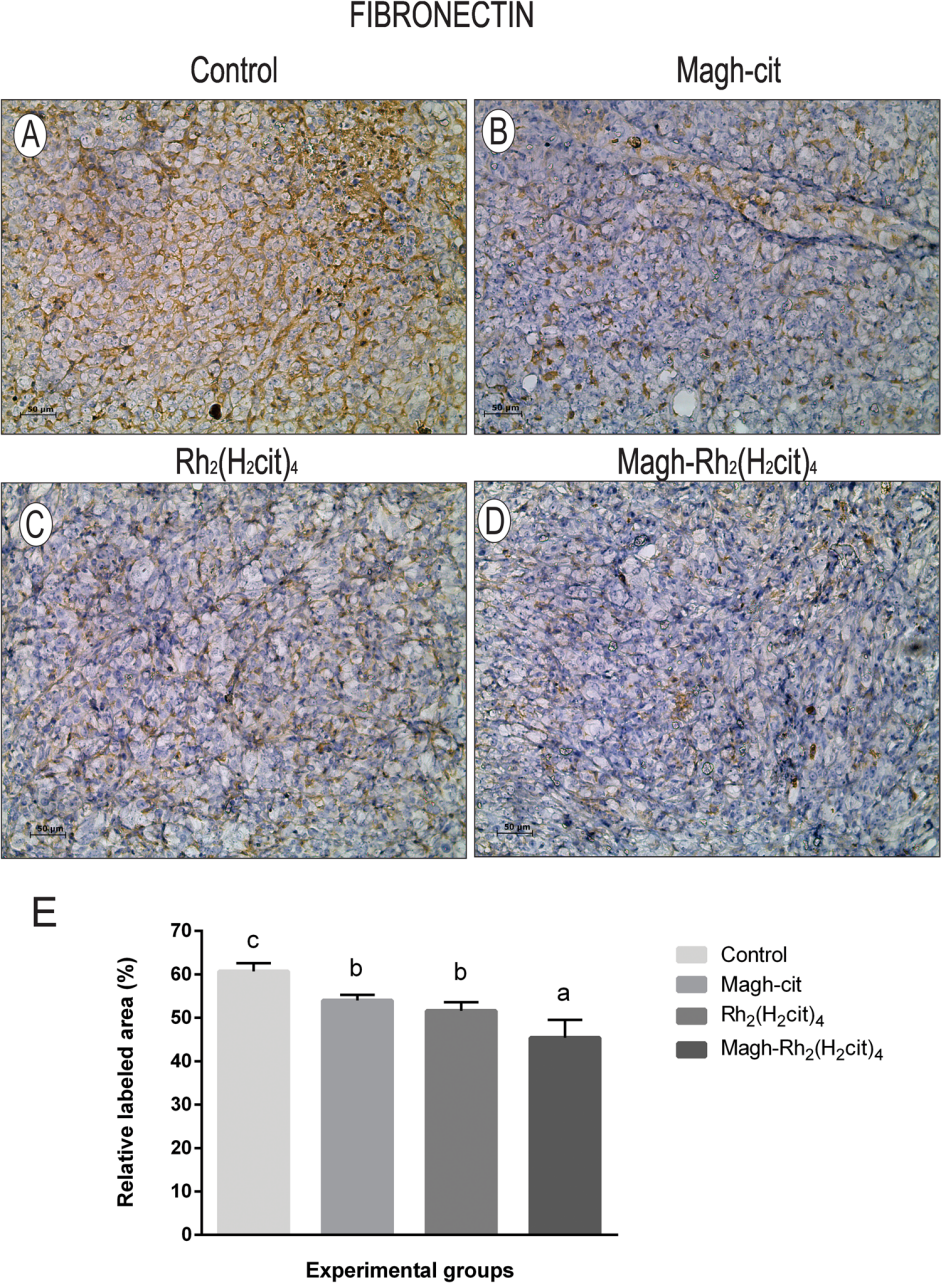
Representative images of fibronectin immunohistochemical detection in tissue of Balb/c mice from each experimental group. (**A**) Control, (**B**) Magh-cit, (**C**) Rh_2_(H_2_cit)_4_, (**D**) Magh-Rh_2_(H_2_cit)_4_, (**E**) Quantification of FN staining. The data represent the average of 10 photos of each group. In Magh-Rh_2_(H_2_cit)_4_ treated animals, the percentage of tissue area occupied by positive cells is significantly reduced compared to the control group mice. (**A–D**) IHC, DAB chromogen, Mayer’s Hematoxylin counterstain, Original magnification 200x. Data represent mean values ± standard error and different letters indicate statistical difference among treatments (p < 0.05). Scale bars: 50 µm.

We observed that the groups which received treatment had a lower expression of FN when compared to the control group (Fig. 4). The group treated with Magh-Rh_2_(H_2_cit)_4_ (Fig. 4D) showed a significant reduction of 25.8% when compared to the control group (Fig. 4A). The groups treated with Rh_2_(H_2_cit)_4_ (Fig. 4C) and Magh-cit (Fig. 4B) displayed a reduction of 14.9% and 11%, respectively, which were not significant when compared with the control group, but when these groups were compared with each other, statistical differences were observed (Fig. 4E). Studies have shown that the expression of fibronectin in breast cancer is greater than in normal tissue, since in tumors there is a marked increase in the expression, and this is related to the aggressiveness of the disease^23^. Another interesting aspect, shown by Gorczyca *et al*.^25^, is the correlation between tumor size and fibronectin expression in breast cancer. The results presented here showed that groups that received treatment and had a significant reduction in tumor volume, consequently, had a significant downregulation of fibronectin expression, according to the expectation.

**Figure 5:**
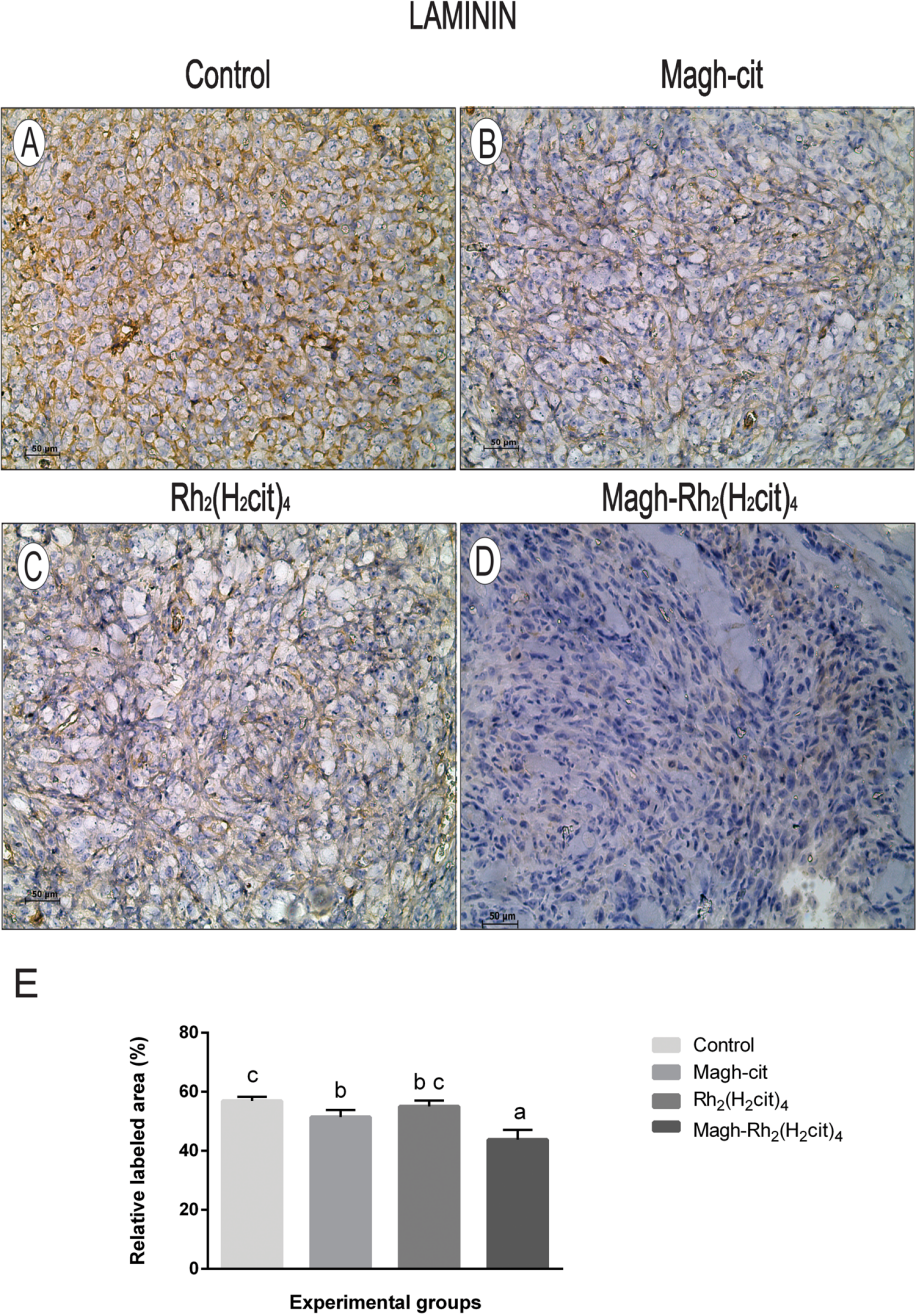
Representative images of laminin immunohistochemical detection in tissue of Balb/c mice from each experimental group. (**A**) Control, (**B**) Magh-cit, (**C**) Rh_2_(H_2_cit)_4_, (**D**) Magh-Rh_2_(H_2_cit)_4_, (**E**) Quantification of LN staining decreased markedly after Magh-Rh_2_(H_2_cit)_4_ treatment when compared to control mice. The data represent the average of 10 photos of each group. (**A–D**) IHC, DAB chromogen, Mayer’s Hematoxylin counterstain, Original magnification 200x. Data represent mean values ± standard error and different letters indicate statistical difference among treatments (p < 0.05). Scale bars: 50 µm.

The results of laminin expression are shown in Fig. 5. A significant reduction of 23% in the group treated with Magh-Rh_2_(H_2_cit)_4_ (Fig. 5D) was observed when compared to the control group (Fig. 5A). Groups treated with Magh-cit (Fig. 5B) or Rh_2_(H_2_cit)_4_ (Fig. 5C) had statistically significant reductions of 9.6% and 3.3% respectively (Fig. 5E). A correlation was observed between tumor size and levels of laminin expression, as observed for fibronectin results. Studies have shown that increased laminin contributes to metastasis and tumor aggressiveness in an *in vivo* experimental model of breast cancer^26,27^. Therefore, we suggest that reduction of fibronectin and laminin expression induced in particular by Magh-Rh_2_(H_2_cit)_4_ treatment is associated with the tumor reduction observed in our results.

### Western blotting analysis

**Figure 6:**
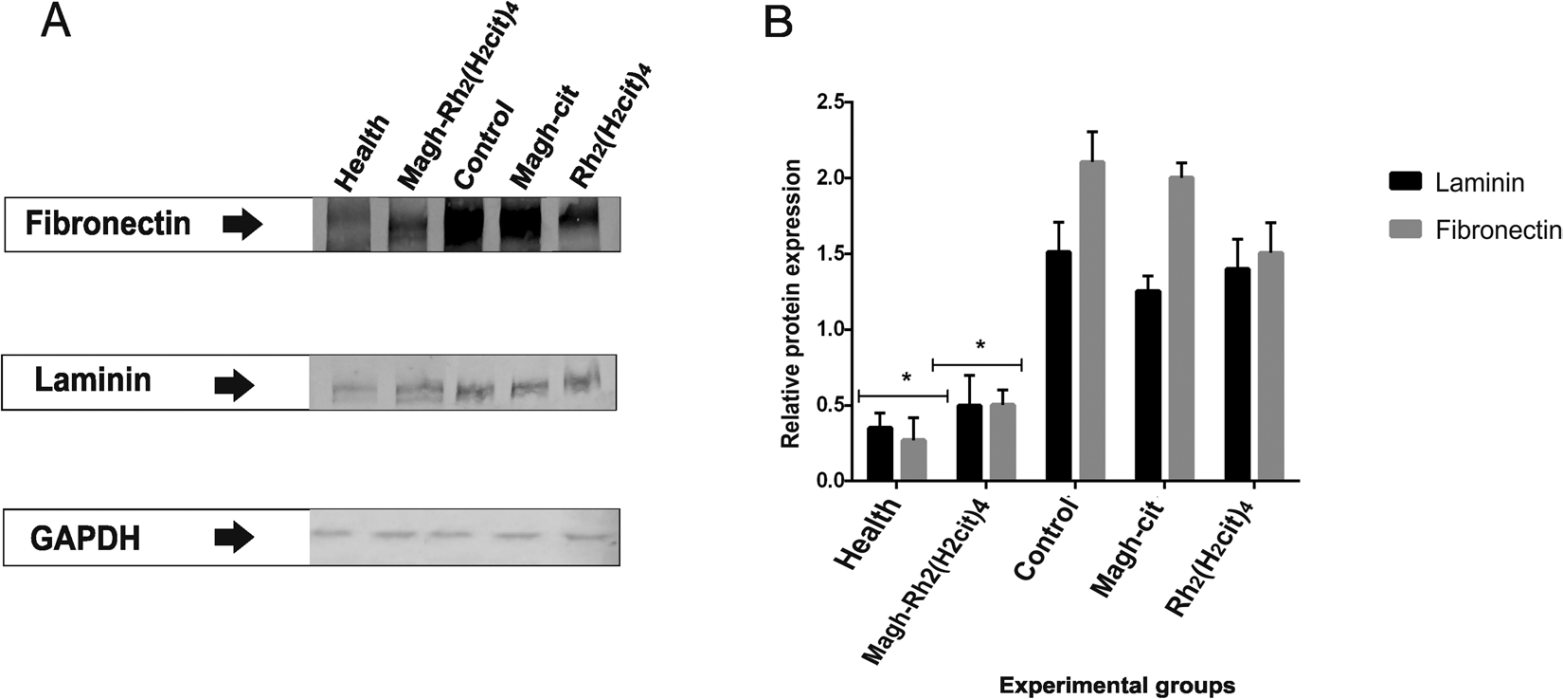
Analysis of FN and LN proteins expression of Balb/c mice bearing 4T1 breast tumor. The animals were treated with Magh-cit or Rh_2_(H_2_cit)_4_ or Magh-Rh_2_(H_2_cit)_4_. (**A**) western blotting analysis shows a band of approximately 250 kDa (FN) and 225 kDa (LN) in the total protein extracts from normal and tumor tissues from mice. (**B**) Quantification of relative protein expression of LN and FN found in western blotting analysis. GAPDH was used as internal control. The healthy and Magh-Rh_2_(H_2_cit)_4_ treated groups showed markedly statistically significant reduction when compared to the control group. The values shown are the mean ± SEM *p < 0.05 and analyzed by an ANOVA; Tukey’s post hoc test.

The western blotting analysis was done as a complementary test to evaluate the FN and LN expression (Fig. 6A). In the control group, we found an increase of FN by 90.4% and by 80% of LN, when compared with the healthy group (Fig. 6B). In the groups that received treatment, the data corroborated with that found in immunohistochemistry. Significant reduction of the expression levels of FN and LN in the group treated with Magh-Rh_2_(H_2_cit)_4_ was 76.1% and 73.3%, respectively, when compared with the control group. One remarkable fact is that the levels in Magh-Rh_2_(H_2_cit)_4_ group are very close to those of the healthy group, and this is probably due to the tumor regression. These results demonstrate a possible ability of the rhodium (II) citrate compound associated with maghemite nanoparticles to function as a modulator of these proteins. It is important to emphasize that the study was done in an animal model with metastatic 4T1 cells, which demonstrates the relevance of the proposed treatment.

A similar study was done by Chaves *et al*.^28^, and their study showed that there does exist a relationship between the increase of FN expression in renal carcinoma and the tumor progression. In addition, it was possible to observe that treatment with the endostatin compound, a potent angiogenesis inhibitor, downregulated the expression of FN in renal carcinoma.

### Real-time PCR (qPCR) analysis

**Figure 7:**
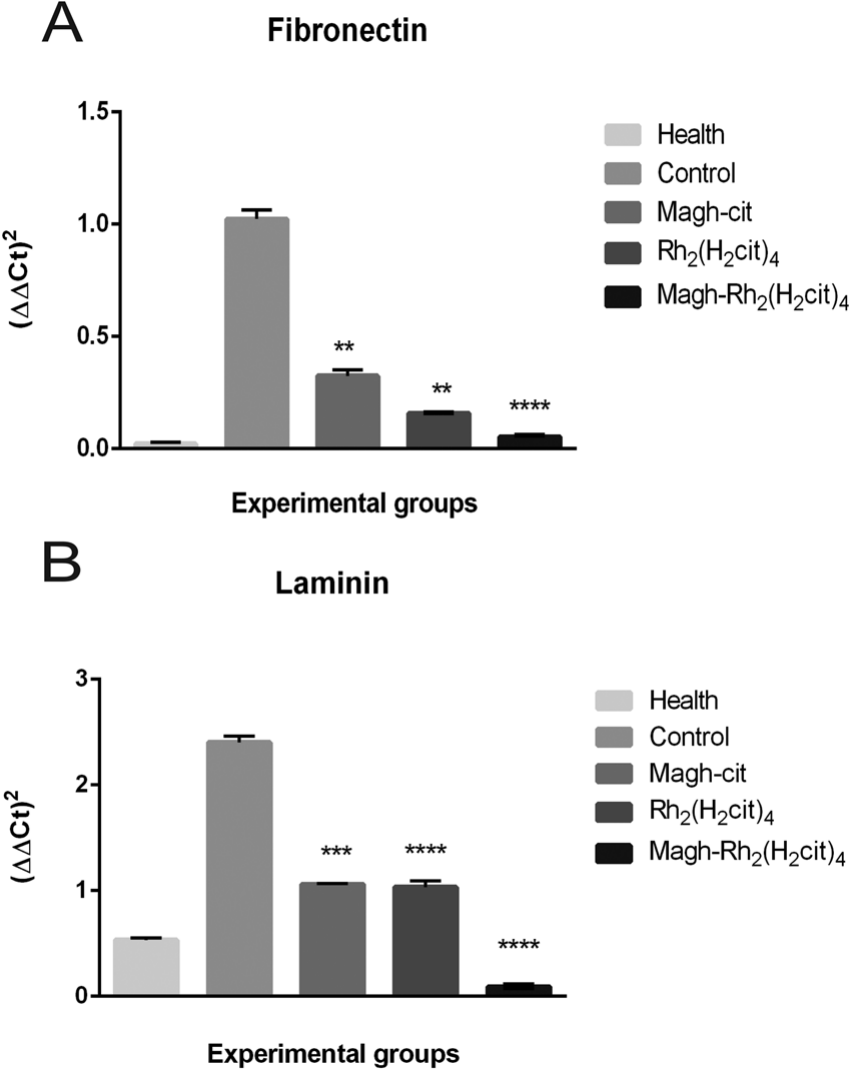
mRNA levels of FN1 (**A**) and LAMA1 (**B**) were measured subject quantitative reverse transcriptase PCR in breast tumor and health breast tissues. Gene expression in breast tumor tissue treated with Magh-cit, Rh_2_(H_2_cit)_4_ and Magh-Rh_2_(H_2_cit)_4_ was compared with control (non-treated) and health group. The cDNA was prepared and 1.5 µg was used for each RT-PCR reaction. Three independent experiments were average. The values shown are the mean ± SEM of three independent experiments in triplicates, **p < 0.01 and ****p < 0.0001.

Our data showed that the expression levels of the FN1 (fibronectin) and LAMA1 (laminin) genes, as seen in Fig. 7, decreased dramatically in response to the treatments used. The fibronectin (Fig. 7A) and laminin (Fig. 7B) transcripts from the Magh-Rh_2_(H_2_cit)_4_ treated group were 95% and 94%, respectively, lower than the control group, and values close to those observed in the healthy group. The analysis revealed that the studied genes had significantly over expressive responses in the control group compared to the levels of transcripts found in healthy tissue and in the treated groups. All groups receiving treatment had a statistically significant reduction of FN1 and LAMA1 when compared to the control. These results open the way to initiate the investigation of the mechanism of action of these compounds in the downregulation of fibronectin and laminin proteins.

Studies have been done to demonstrate the relationship between the expression of the FN1 and LAMA1 genes with the ability to invade various types of tissues, including breast, prostate and ovaries among others^29^.

FN1 expression has been proposed as a molecular marker to identify malignant lesions. A study done with 86 thyroid carcinoma patients investigated the levels of some genes among them, FN1. An overexpression of FN1 in tumor tissues was observed when compared to non-tumor tissues. Among those studied, the FN1 gene was the gene that most confers aggressive characteristics and plays an important role in the progression of tumors^30^.

In conclusion, our results suggest there is an overexpression of the fibronectin and laminin proteins in mice bearing metastatic breast tumors. Treatments based on Magh-Rh_2_(H_2_cit)_4_ and Rh_2_(H_2_cit)_4_ compounds significantly reduced tumor volume, and did not cause adverse effects in the animals. Consistently, the techniques used showed that treatments also downregulate the expression of FN and LN proteins, correlate with the level of expression of these proteins, and are directly related to tumor progression. Moreover, treatment using nanoparticles as drug carriers promotes better antitumor results and could be considered as a promising alternative to traditional treatments.

## Experimental procedures

### Characterization

The nanoparticles were characterized using various techniques such as transmission electron microscopy (TEM), X-ray diffractometry (XRD), energy dispersive spectroscopy (EDS) and dynamic light scattering (DLS). The morphology and size of nanoparticles were obtained by using a JEOL JEM-1110 TEM operated at 100 kv. Samples of Magh-cit and Magh-Rh_2_(H_2_cit)_4_. Samples of Magh-cit and Magh-Rh_2_(H_2_cit)_4_ were diluted at appropriate concentration and then dropped on grids (400 mesh) coated with Formvar film and dried in ambient temperature. The particles size distribution was determined by at least 400 samples counts. X-ray diffraction (XRD) pattern was performed to identify the crystal structure of nanoparticles and was obtained using Rigaku (Miniflex 600) Cu Kα (λ = 1.54 Å) radiation scanned for 2θ. The crystallite sizes of γFe_2_O_3_ (311) phase were determined using the Scherrer formula. The elemental analysis was performed by energy-dispersive X-ray spectroscopy (EDS, JEOL, JSM 7001 F). The zeta potential was measured by electrophoretic mobility using a ZetaSizer Nano (ZS- ZEN3600, Malvern instruments, UK). Samples (0.1 ml) were diluted 100 times using double phosphate buffered saline. The analysis was performed in triplicate. To analyze how samples, behave at different storage temperatures (4 °C and 37 °C), heating-cooling cycles were carried out. Samples of nanoparticles were collected into an eppendorf tubes and were subjected initially to 4 °C in refrigerator by 24 hours and then the same sample was heated for 24 hours in incubator at 37 °C, thus completing a cycle. After the end of each cycle, the samples were diluted in phosphate buffered saline and the hydrodynamic diameter, zeta potential and PDI were measured on the ZetaSizer equipment. Each sample was submitted to six cycles.

### Syntheses of Maghemite nanoparticles and Rhodium citrate

Maghemite (γ-Fe_2_O_3_) nanoparticles and maghemite nanoparticles functionalized with rhodium (II) citrate (Magh-Rh_2_(H_2_cit)_4_) were prepared according to procedures described previously^31^.

The synthesis of Maghnetite (Fe_3_O_4_) nanoparticles were made by mixing FeCl_2_ and FeCl_3_ aqueous solutions (2:1 molar ratio) with NaOH solution. The solid was washed with distilled water until pH 9 and oxidation of magnetite to maghemite was performed adjusting the pH to 3, stirring the dispersion under heating and constant oxygen flow. After that was centrifuged, dispersed in water, and dialyzed for 24 hours.

For coating maghemite nanoparticles with rhodium citrate, 5 mL of the magnetic dispersion and 1 mL of rhodium (II) citrate solution (0.054 molL^−1^) were mixed and stirred for two hours at room temperature. The nanoparticles were separated by centrifugation (5000 rpm), washed (3x) with deionized water and dispersed in 5 mL of water. The stable magnetic solution containing Magh-Rh_2_(H_2_cit)_4_ nanoparticles was obtained by adjusting the pH to 7.

### Cell line

The murine breast tumor cell line, 4T1 (Rio de Janeiro cell Bank, BCRJ, Brazil), was maintained in Dulbecco’s modified Eagle’s medium (DMEM, GIBCO) supplemented with a 10% Fetal Bovine Serum (FSB, GIBCO) and a 1% penicillin-streptomycin (GIBCO) in a humidified chamber at 37 °C with 5% of CO_2_.

### Animals

All animal experiments were done in accordance with the standards of the Biological Science Institute under a protocol that was approved by the Animal Research Ethics Committee of the University of Brasilia (number of process: 329781/2013), Institute of Biological Science, Brazil. Thirty female Balb/c mice (six in each experimental group) weighing 17 to 20 g and 12 weeks old were obtained from the Laboratory Animals Breeding Center (Cecal), Fiocruz, Rio de Janeiro, Brazil. The animals were maintained on free access to food and water, and under conditions of 12 h dark/light cycles.

### Tumor inoculation

Animals were anesthetized with a ketamine (80 mg/kg body weight) and xylazine (10 mg/kg body weight) solution. Then 2 × 10^4^ 4T1 cells (suspension of 50 µL serum-free DMEM) were inoculated in the abdominal mammary gland region. Seven days after inoculation of 4T1 cells, the mice were separated into five groups of six mice in each group. Tumor length (L) and width (W) were measured every day using a digital caliper, and tumor volume was calculated using the following formula: Tumor volume = *0,4 (L* × *W* ^2^) according the literature^32^.

### Treatments

Before treatment, all mice were weighed and identified. All mice from the treatment and control (non-treated) groups developed tumors. The weight and tumor volume of each mice were measured over a period of 21 days. Intra-tumor injections (50 µL) were carried out every two days, totaling five applications. The experimental groups and the treatments used are described below:

- (Group 1) - animals treated with rhodium (II) citrate associated with maghemite nanoparticles [Magh-Rh_2_ (H_2_cit)_4_] with 8 mg/kg rhodium citrate and 216 mg/kg iron;
- (Group 2) - animals treated with free rhodium (II) citrate [Rh_2_ (H_2_cit)_4_] with 8 mg/kg;
- (Group 3) - animals treated with maghemite nanoparticles coated with citrate [Magh-cit] with 216 mg/kg iron;
- (Group 4) control group with tumors were treated with physiological saline solution (rhodium (II) citrate solvent);
- (Group 5) animals without tumor cell transplantation (healthy), were submitted to injections containing a 0.9% physiological saline solution in the mammary gland region, to maintain the same experimental conditions.

Mice were treated with Rh_2_ (H_2_cit)_4_, Magh-Rh_2_ (H_2_cit)_4_ and Magh-cit with doses prepared according to the body mass of each mouse, resulting in equal concentrations of rhodium citrate and Iron per kilogram of body mass. The rhodium compound and Iron oxide were used with the defined concentrations for each of the five applications administered, as described in Table 3.

**Table 3:** Rhodium (II) citrate and iron (II) oxide concentrations of each treatment used during the experiment.

Compounds	Dose (mg/kg)	
	Rh_2_(H_2_cit)_4_	Fe_2_O_3_
Rh_2_(H_2_cit)_4_	8	0
Magh-Rh_2_(H_2_cit)_4_	8	216
Magh-cit	0	216

### Euthanasia

On the 22nd day after tumor cell transplantation (one day after the last treatment injection), mice were anesthetized by cervical dislocation, weighed, and submitted to blood collection. Tumors and the breast were collected and half of the tumor was fixed in a 4% paraformaldehyde solution. The other half was frozen (80 °C).

### Protein extraction and Western Blotting analysis

All proteins from tumors and breast samples were extracted using TissueLyser equipment (Qiagen) with 500 µl of extraction buffer (Tris-HCl 50 mM at pH 8, NaCl 150 mM, Triton × 0.5%, MgCl_2_ 1 mM) at 5 hertz for 5 minutes. The supernatant was collected and stored at −80 °C. Protein concentration was measured using a Bicinchoninic acid (BCA) assay kit in accordance with the manufacturer’s instructions, using bovine serum albumin as the standard protein.

SDS-Polyacrylamide gel electrophoresis was treated with non-reducing conditions. Samples (20 µg of protein) were run on a 7.5% SDS-PAGE gel. Samples were separated at 100 Volts for 1.5 h. After, the proteins that were on gel were transferred to nitrocellulose membranes at 15 Volts, overnight at 4 °C, under stirring.

Then, the membranes were blocked with TTBS buffer/milk (TBS, 1% Tween 20, 5% non-fat dry milk) for 1 hour, washed with TBS, and incubated with primary antibodies for fibronectin (Abcam, ab23750, 1:500), laminin (Abcam, ab11575, 1:500) and GAPDH (Abcam, ab8245, 1:10000) in a horizontal shaker at 4 °C. After being washed three times with TBS, the membranes were incubated with goat anti-mouse or anti-rabbit IgG conjugated to Alkaline Phosphatase (whole molecule) secondary antibody (Sigma Aldrich, A3562 or A3687, 1:10000) in TTBS for 1 hour. The membranes were then washed three times and the protein was stained with NBT (nitroblue tetrazolium chloride) and BCIP, (5-bromo-4-chloro-3-indolyl-phosphate, toluidine-salt) a colorimetric detection system. The reaction was stopped by washing with distilled water. Three independent experimental repeats were performed. The Western blot signal was normalized to the GAPDH band density. Bands were quantified using, Image J software.

### Sample preparation

For immunohistochemical analyses, the tumors and breast tissues were fixed in paraformaldehyde (4%) in a phosphate buffer (0.1 M, pH 7.4) and dehydrated in serial baths of increasing concentrations of ethyl alcohol (70–100%). Then, they were diaphanized with xylene and embedded in paraffin. The process was done with the aid of an automatic tissue processor (Oma DM-40, São Paulo, Brazil). Histological sections of 3 µm thickness of tumors and breast were obtained serially, discarding five sections between each on Leica microtome (RM 2235, Germany).

### Immunohistochemistry

Fibronectin and laminin proteins were immunolocalized using the immunohistochemical method. The slides with histological sections were deparaffinized and rehydrated. For antigen retrieval, slides were immersed in a citrate buffer (3 mM, pH 6.0) for 20 minutes at 120 °C. After that, slides were washed three times in TBS (15 min each). In order to inhibit endogenous peroxidases, slides were incubated (twice) in a 3% solution of hydrogen peroxide (15 minutes each) at room temperature. The non-specific binding sites were blocked with 3% BSA for 1 hour at room temperature. Then, slides were incubated with primary antibody anti-fibronectin (Abcam, ab23750, 1:50) or anti-laminin (Abcam, ab11575, 1:50) diluted in 3% BSA overnight at 4 °C, washed and then incubated with biotinylated secondary antibodies (Streptavidin-Peroxidase) for 30 minutes at room temperature. Slides were washed and incubated with a working solution of diaminobenzidine (DAB) for 10 seconds and counterstained with Mayer’s Hematoxylin.

### Immunohistochemistry quantification

Fibronectin and laminin immunohistochemical labeling were quantified by digital image analysis according to the procedure described by Ricci *et al*.^33^ with modifications. The process consisted of using the batch area measurement function of the ImageJ software, after application of an adequate threshold with a macro plugin that was individually adjusted for each of the two labeling types^34^. For each extracellular component (fibronectin or laminin), ten micrographs at 200x magnification were taken of randomly selected non-contiguous areas for each section (three sections from each of three animals per treatment) by using a Zeiss Axiophot microscope equipped with a Zeiss MC 80 DX digital camera, which produced homogeneous 24-bit color images with a 1,024 × 768-pixel size and 150 dpi resolution. The mean of the relative area labeled (%) measured from each set of ten microscopic fields was used to generate a single value for each section.

### Real-time PCR

RNA from breast and tumor tissue was extracted using TissueLyser equipment with Trizol reagent (Life Technologies Inc., Rockville, MD, USA) according to the manufacturer’s protocol. The RNA concentration was determined using a Nanodrop spectrophotometer ND-100 (Thermo Fisher Scientific Inc.1, Waltham, MA). Samples were stored at −80 °C until analysis.

Real-time PCR analysis was performed in triplicate, in an Applied Biosystems 7300 Real-time system. Expression levels of the FN1 and LAMA1 gene were performed with specific TaqMan probes (Applied Biosystems). The glyceraldehyde 3-phosphate dehydrogenase (GAPDH) was used as an internal control (Table 4). Cycle conditions were as follows, using a decontamination stage for an initial 2 min at 50 °C and 10 min at 95 °C enzyme activation stage, the samples were cycled 40 times at 90 °C for 15 s melting stage and 60 °C for 1 min in annealing stages. Statistical analysis of the results was performed using one-way ANOVA, with p < 0.05 as the minimal level of significance

**Table 4:** Primer and probe acquired from Applied Biosystems.

Gene name	Assay ID	Symbol
Fibronectin	Mm01256734_m1	FN1
Laminin	Mm00439445_m1	LAMA1
GAPDH	Hs02786624_g1	GAPDH

### Statistical analysis

All statistical analyses were performed using commercially available statistical software, GraphPad Prism 6.0. Data were analyzed by one-way analysis of variance (ANOVA) with Tukey’s post-hoc tests and p < 0.05 was considered statistically significant.

### Data availability

All data generated or analyzed during this study are included in this published article (and its Supplementary information files).

## Electronic supplementary material

Supplementary Information
