# Supplementary material for: Modulation of fibronectin and laminin expression by Rhodium (II) citrate-coated maghemite nanoparticles in mice bearing breast tumor
Source: Sci Rep. 2017 Dec 20;7:17904. doi: 10.1038/s41598-017-18204-1 (PMC5738373; doi:10.1038/s41598-017-18204-1)
Supplement: Supplementary file 1 — Supplementary Information [file 41598_2017_18204_MOESM1_ESM.pdf]

Modulation of fibronectin and laminin expression by Rhodium (II) citrate-coated maghemite nanoparticles in mice bearing breast tumor

Márcia Rocha[a], Rachel Arcanjo[b], Cláudio Lopes[b], Marcella Carneiro[b], Aparecido Souza[c] and Sônia Báo[b,*]

Supplementary information

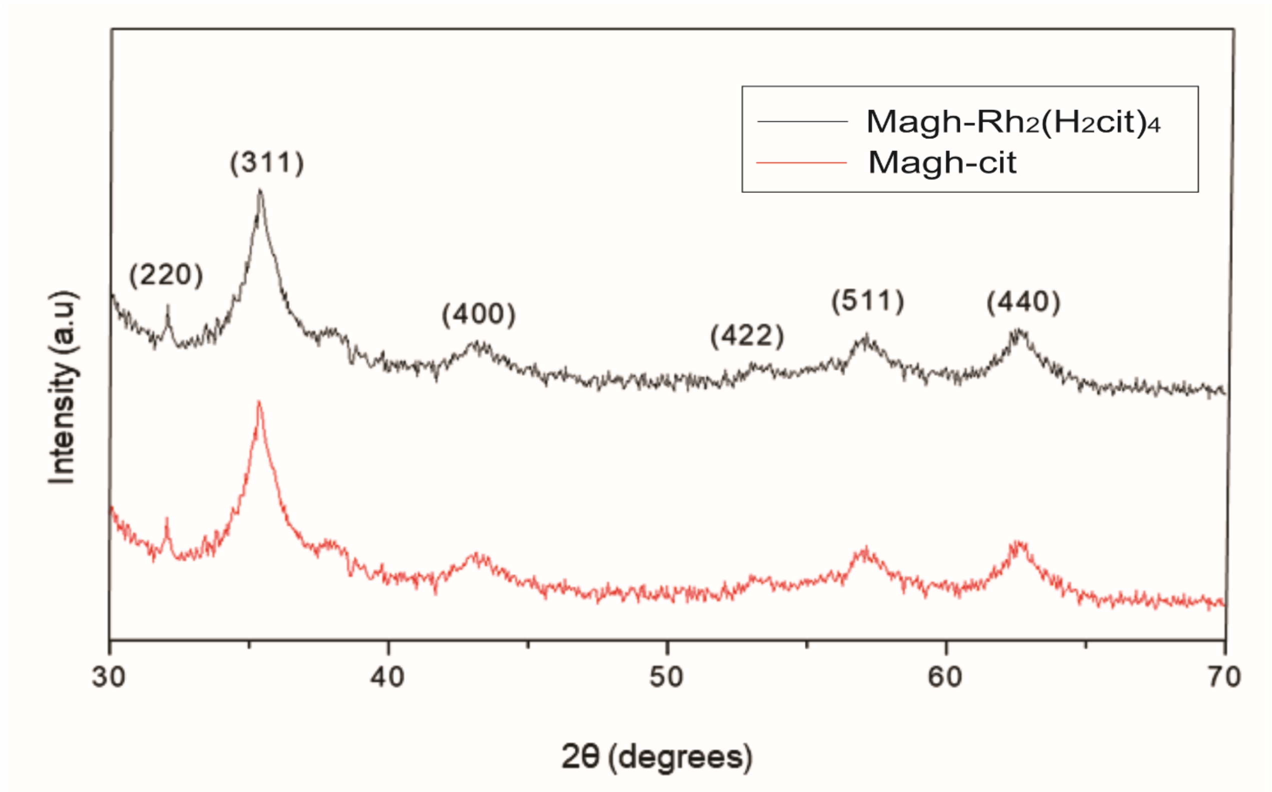

Figure S1: X-ray diffraction (XRD) diffractogram patterns of Magh-cit and Magh-$Rh_2(H_2cit)_4$.

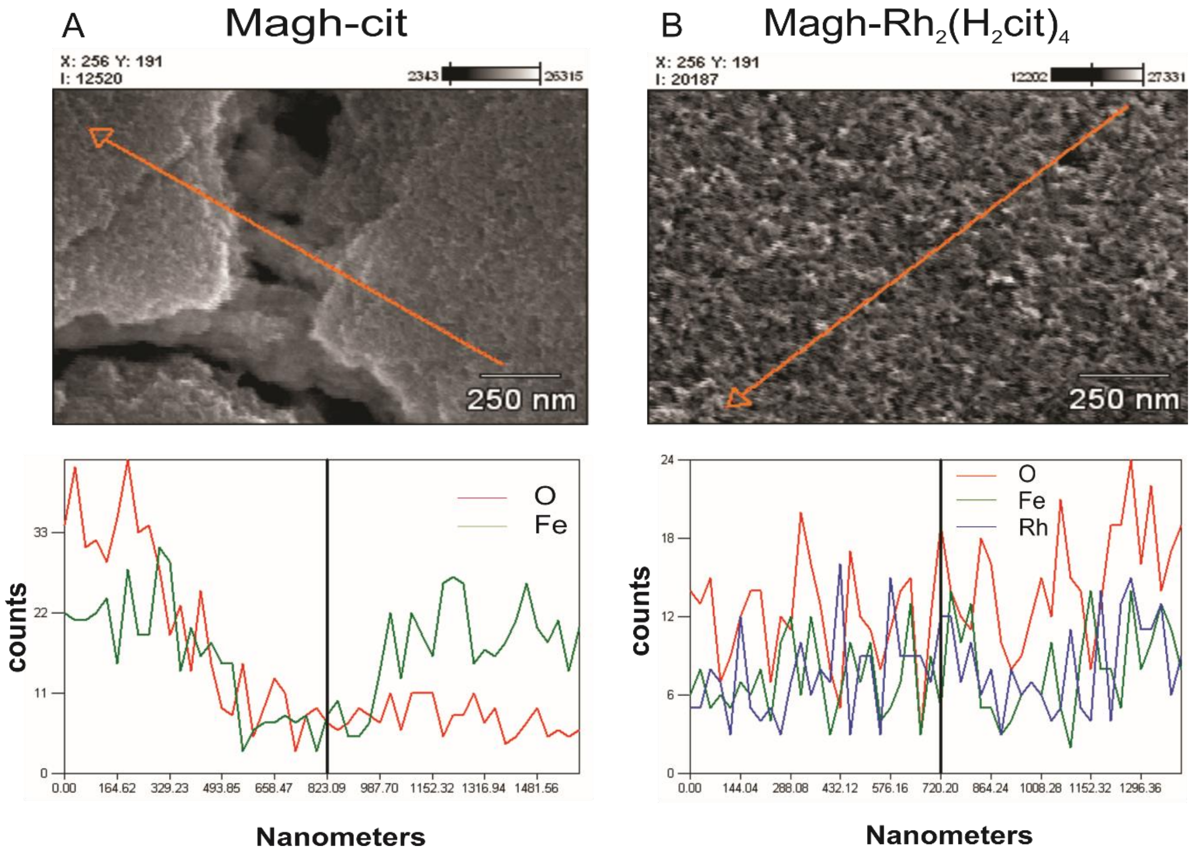

Figure S2: Energy dispersive spectra (EDS) of Magh-cit (A) and Magh-$Rh_2(H_2cit)_4$ (B) nanoparticles.

Supplementary table 1: Biochemical and hematology parameters of female Balb/c mice bearing breast carcinoma treated intratumorally with $Rh_2(H_2cit)_4$, Magh-$Rh_2(H_2cit)_4$ and Magh-Cit.

**Parameters**	**Health**	**Control**	**$Rh_2(H_2cit)_4$**	**Magh-$Rh_2(H_2cit)_4$**	**Magh-Cit**
Biochemical parameters					
**Creatinine (mg/dL)**	0.2 ± 0.1	0.3 ± 0.0	0.3 ± 0.1	0.2 ± 0.0	0.2 ± 0.0
**ALT (U/L)**	38.3± 1.5	39 ± 1.0	38.2 ± 1.2	37.4 ± 2.6	37.6 ± 2.5
Hematology parameters					
**HGB (g/dL)**	14.0 ± 0.4	12.8 ± 0.5	10.7 ± 2.3	12.7±1.0	13.4 ± 0.2
**HCT (%)**	42.9 ± 0.3	39.6 ± 0.2	42.1 ± 0.3	41.0 ± 0.2	42.3± 0.4
**MCHC (g/mL)**	32.6 ± 0.4	32.3 ± 0.3	30.1 ± 1.5	31.0 ± 1.4	31.7± 0.2
**MCH (pg)**	15.7 ± 0.2	15.3 ± 0.1	14.5 ± 0.7	15.1± 0.7	15.5 ± 0.3
**MCV (fL)**	48.3 ± 0.2	47.3 ± 0.3	48.2 ±0.3	48.9 ± 0.4	48.8± 0.7
**WBC (/mm3)**	3816±4057	2040±3958	3352±1009	3654±1810	3866±3958
**Lymphocytes (%)**	40.2 ± 2.1	44.8 ± 3.1	41.2 ± 2.3	43.0 ± 3.3	46.0 ± 2.2
**Eosinophils (%)**	5.6 ± 1.2	5.4 ± 0.9	5.2 ± 0.7	4.0 ± 0.40	4.9 ± 0.32
**Monocytes (%)**	48.3 ± 4.6	46.9 ± 4.2.0	47.7 ± 2.0	47 ± 2.8	3.3 ± 6.9

Legend: ALT- Alanine Transaminase; RBC - Red Blood Cells; HGB - Hemoglobin; HCT Hematocrit; MCHC - Mean Corpuscular Hemoglobin Concentration; MCH - Mean Corpuscular Hemoglobin; MCV- Mean Corpuscular Volume; WBC - White Blood Cells; Data correspond as mean ± standard error.
